# Predictors of long-term care use - informal home care recipients versus private and public facilities residents in Poland

**DOI:** 10.1186/s12877-023-04216-2

**Published:** 2023-08-24

**Authors:** Małgorzata Wrotek, Małgorzata Kalbarczyk

**Affiliations:** Faculty of Economic Sciences, University of Warsaw, Długa 44/50, Warsaw, 00-241 Poland

**Keywords:** Long-term care, Informal care, Poland, Andersen's behavioral model

## Abstract

**Background:**

The population aging, together with the shrinking caring potential of families, is a major challenge for social policy in the coming years. The aim of the study is to identify the factors that determine not only the use of long-term care (LTC) but also the selection of individual types of such care in Poland.

**Methods:**

Using unique data collected from inpatient LTC facilities in Poland and the Survey on Health, Ageing and Retirement in Europe (SHARE) database, we estimate logistic regressions explaining the choice of LTC solution.

**Results:**

Our results suggest that social inequalities play a role in choosing the type of LTC. Better educated people choose private institutions, while people without support network use more often social residential homes. The impact of multimorbidity on choosing different types of inpatient facilities is limited, thus the number of ADL limitations remains a better indicator of long term care utilization.

**Conclusions:**

The study confirms that social inequalities influence decisions about the choice of LTC. However, multi-morbidity is a predictor of using LTC to a limited extent. The differences in LTC selection determinants between women and men are noticeable.

**Supplementary Information:**

The online version contains supplementary material available at 10.1186/s12877-023-04216-2.

## Introduction

In the last 20 years, the percentage of people aged 65 and over increased in the EU-27 by 5.4 pp. reaching 24.6% in 2021^1^. In Poland, despite the fact that this indicator was lower than the average for the EU countries – 21.4%, it grew at an even greater rate of 6.5 pp [1]. The EUROPOP-19^2^ forecasts also show that in 2060 the increase in the percentage of people aged 65 + in Poland, as compared to 2021, will be more than twice as high as the average increase for the EU-27 (12.5 pp. vs. 5.7 pp.), and in 2070 Poland will see the highest growth of this indicator among all the EU countries [2]. In turn, the percentage of people aged 80 and over living in Poland in 2021 was 4.4% [1]. And although this value was lower than the EU-27 average (6%), Poland was among the 12 countries where the fastest growth of this indicator was noted over the last 20 years. By 2030, the increase in the percentage of people aged 80 and over for Poland will be higher than the average for the EU-27 countries. In 2070, the share of this age bracket in the total population will reach 15.6%, which means that Poland will experience the highest growth (11.2 pp.) in comparison with all EU countries [2].

The aging of the population increases the demand for LTC services. According OECD [3], LTC is defined as the services provided to persons dependent on activities of daily living (ADL) [4] and instrumental activities of daily living (IADL) [5] for an extended period of time and it may be provided in nursing homes, in assisted living facilities, in the community or at home [6]. As the number of older adults dramatically increases, it becomes a challenge for public policy in both the delivery of LTC services and expenditure on LTC. Thus, the progressive aging of the population makes us reflect on the factors leading to the choice of specific forms of LTC. In our study we use Andersen’s Behavioral Model of Health Services Use (1968) [7] to investigate how particular characteristics of the older adults correlate with using different forms of inpatient and informal care.

The aim of our study is to identify factors influencing the use of LTC and the selection of specific forms of residential care in relation to informal care in Poland. According to our knowledge, this is the first study of this type, presenting a quantitative approach based on data from Poland, as well as the first study involving three different types of inpatient LTC facilities, especially still poorly researched private inpatient sector.

In post-communist countries such as Poland, there is high supply of informal care and low supply of formal care [8, 9]. The tendency to use residential care remains low [10] and the caring functions are mainly performed by the family [11], which suggests that cultural factors shape caring patterns. However, with declining caring potential of families, there is increasing pressure to develop formal forms of LTC. LTC in Poland includes cash and in-kind benefits and is provided by the health care, social assistance and private sectors. Two levels can be distinguished [12, 13]: formal (institutional) care provided at home or in inpatient facilities and informal care (informal caregivers, most often family members). In terms of inpatient care in Poland, as of December 31, 2020, there were 30,638 people in long term care health sector facilities^3^ [14], 18,176 people in officially registered private rest homes and 75,133 people in social residential homes [15]. At the same time, the total population of Poland was 38.1 million, of which 7.1 million were aged 65+ [16].

The criteria for admission to care facilities and the way in which they work are regulated by the relevant legal acts in Poland [17, 18]. Both residential social homes and private rest homes are intended for persons who require 24/7 care due to age, illness or disability, who are unable to function independently in daily life and for whom the necessary care cannot be provided at home. Where these people also require enhanced medical care, they are referred to nursing homes. During admission to LTC facilities, documents are required to prove the health status and income situation of the potential resident/patient. In the case of residential social homes and private rest homes, a medical certificate of the health status of the person applying for admission is required, while in the case of nursing homes, Barthel scale scores and health insurance are additional criteria. The amount of fees varies regionally. In the case of private rest homes, the cost of the stay is paid in full by the residents (and/or their family). The stay in residential social homes and nursing homes is also chargeable, but the residents pay no more than 70% of their income. In the case of residential social homes, if the resident is not able to pay the fee himself, the spouse and children are obliged to do so, and if this is not enough, the municipality then contributes to the costs. In nursing homes, the fees paid by the patients (and/or their families if they have previously agreed to contribute to the costs) cover the costs of accommodation and meals, with the remaining amount being covered by the National Health Fund [17, 18].

In Poland, LTC remains underfunded compared to the countries of Western and Northern Europe, as the expenditures on LTC (as % of GDP) remain relatively low. In the coming years, with the progressive aging of the population, the pressure on their growth is expected to increase. Additionally, solutions used in Poland, based on universal and wealth-related systems [19], mean that access to various forms of residential care is not equal and socio-economic factors seem to play an important role in both decisions related to the choice of LTC form, and in health inequalities.

In our study, the following research hypotheses will be verified:

1. Social inequalities play a role in long-term care decision-making.
2. Multi-morbidity (number of chronic diseases) is not a good predictor of LTC use.
3. There are different patterns of long-term care utilization between females and males.

### Theoretical and empirical issues

Andersen’s Behavioral Model of Health Services Use, although originally used to predict the use of healthcare services, is now also used extensively in research focusing on actual LTC use. The original version of the model from 1968 focused on the family as the unit of analysis [7] and listed 3 groups of factors: predisposing, enabling and need as individual and contextual determinants of the use of healthcare services [20]. However, difficulties in developing measures at the family level led to the evolution of this model towards the patient as a sole decision-making entity [21]. In the following years, extensions were introduced to the original model, taking into account e.g. variability of individual factors over time, factors related to the health care system, measures of use of health services or consumer satisfaction as well as additional variables related to the external environment, making the model a useful tool for health policy or health reforms [21].

The explanation of the importance of the main factors (in relation to healthcare for which the original model was developed) was extensively described by Andersen and Davidson [22], where: (1) the term predisposing factors at the individual level refers to demographic characteristics of age and gender, social, i.e. education, profession, ethnicity or social relations, e.g. related to family status, mental factors, i.e. health values, attitudes towards health or knowledge related to health. In terms of the contextual dimension, predisposing factors are, *inter alia*, demographic and social composition of the population, cultural norms, organizational and collective values, political factors; (2) the term enabling factors refers to the group of factors enabling the use of services, i.e. financial factors (e.g. income, assets, price of healthcare services) and organizational factors (e.g. having a regular source of care and its nature, waiting time for care). From the contextual perspective, enabling factors of a financial nature will therefore refer to e.g. income per capita, the relative price of goods and services, expenditure on health care, and in terms of organization to e.g. the type, structure, location, number and distribution of health facilities and personnel, education and information programs, or health policies; (3) the term need factors refers to health status, functional status and disease symptoms at the individual level, and to environmental needs or population health indicators at the contextual level.

The determinants of LTC utilization based on the original version of the Andersen’s model or its extension was widely studied [23–35]. These models were used in the context of utilization [31, 33, 36] or transition [27, 34] and both in terms of actual data [24, 33] or intended data [24, 31]. Some of the studies focused on applying the model to informal care [27] or home and community LTC [25–27, 29, 31, 32], while others focused also on institutional inpatient care [29, 31]. Many of these studies built on the original division into predisposing, enabling and need factors. However, there are also numerous other studies focused on the determinants of LTC utilization, even though they were not formally based on the Andersen’s model. In many studies, the need factors were classified in the same way, but there were differences in the classification of the predisposing and enabling factors, as the caring potential of families (e.g. number of children or family contact frequency) was mentioned most often among enabling factors.

#### Predisposing factors

Many studies confirm the positive relationship between age and LTC demand [27, 30, 31, 33, 36–38]. However, the relationship between age and the demand for LTC is not obvious, as some studies showing a positive correlation between age and institutional LTC do not include variables relating to the level of dependency. In studies of the American population over 70 years of age, variable time to death (TTD) proves to be a significant factor in increasing the use of institutional LTC. However, the availability of informal caregivers, especially spouses, significantly reduces this effect [39]. Wren et al. [40] show that the convergence between female and male life expectancy, caused by faster male life extension, significantly contributes to falling demand for both health care and LTC.

Gender remains an important factor influencing the propensity for and use of LTC, but the results are inconsistent. Some findings show a higher probability of using LTC services among females than males [27, 30], mostly explained by their longer average life expectancy compared to males [41–43], as well as chronic diseases (which cause a decline in functional abilities) occurring more severely in this group [44], or a higher probability of experiencing loneliness at the end of life [45]. However, in the literature opposite results can also be found, i.e. a greater risk of institutionalization of males than females, which is most often explained by the greater difficulties with daily chores among males [34].

The relationship between the level of education and morbidity [46] and mortality [47–49] has been addressed in numerous studies. Among better educated people, there is a higher probability of staying in good health [50] and less interest in inpatient LTC [37]. Although there is also evidence in support of an alternative concept [51, 52]. Better education is associated with greater knowledge about the availability and possible types of formal care [51], which leads to increased use of formal home care and reduced informal care [52], or the choice of private care and reduced public care at the same time [53] by better educated people.

#### Enabling factors

Taking into account the structure of households, it is indicated that the risk of using formal LTC increases when living alone [54, 55]. Living with a spouse or daughter reduces the demand for institutional LTC to a greater extent than living with other relatives [56, 57]. However, when medical needs increase, the fact of having a spouse does not translate so clearly into a reduced need for inpatient care [58]. A Canadian study comparing the patient profile of LTC nursing homes with retirement homes shows that people with a spouse predominate in the first type of facilities, while single people in the second type [58]. Not only having children but also close relationships (frequency of visits etc.) with children play an important role in the LTC utilization patterns. According to some findings, when community care is compared with, respectively, home and institutional care, it turns out that older people who have a closer relationship with children are more likely to stay at home, and people who had less frequent of contacts are more likely to opt for institutional care [26].

Older people with higher incomes less frequently use institutional LTC [54, 55, 59–61] because they are able to pay more for additional home care [62]. Inpatient care remains a relatively inferior option when home care is affordable [62–65]. However, when comparing the informal to the formal, higher income increases the odds for utilization of formal LTC care [27], but also the first time LTC services utilization risk has been found to be lower among households with higher gross income [30]. There is also evidence of fairly limited impact of the income level on LTC utilization patterns [26]. Among the wealthy older adults, especially those with real estate, the lower risk of using institutional LTC may be explained by the increased efforts of relatives to inherit their property [66].

The place of residence is also relevant. People living in rural areas have a lower risk of being beneficiaries of institutional LTC than those living in urban areas [31, 67]. It might be explained by different patterns of care between urban-rural areas, especially when seniors living in the village receive more help from family members than inhabitants of large cities [68].

#### Need factors

The morbidity and dependence that accompany the progressive aging processes are mentioned as the main determinants of the demand for formal LTC. The patterns of dependence and morbidity may, however, be different in particular countries, which is explained in the hypotheses existing in the literature: expansion of morbidity [69–71], compression of morbidity or disability [72], dynamic equilibrium, which combines the elements of both the expansion and compression hypotheses [73], or the concept of healthy aging [74]. Environmental changes and medical progress may make living with a disease less burdensome [75–77], while greater care for one’s own health may contribute to a decline in disability among the older adults [78].

The presence of an additional chronic disease increases the probability of utilizing any kind of LTC services [30] or institutional LTC [26] but there is also evidence of the insignificance of this variable for the risk of either home or institutional care [29]. The coexistence of several chronic diseases (multi-morbidity), especially dementia, Parkinson’s disease, urinary incontinence, and fractures as a result of falls, shows a positive correlation with ADL limitations and the demand for institutional LTC [38, 45, 59, 79–83]. However, some studies [37] distinguish between dependency which is measured with ADL limitations (related to the demand for residential care) and IADL limitations (which determines the use of formal home care mainly), where the first indicator (named ADL limitations or disability or dependency levels) is recognized as one of the most important predictors of LTC use [31], especially nursing facilities [35].

### Data and methods

In the presented study, we combined two databases: data from LTC facilities collected by us and available data from SHARE. We decided to combine the data from both databases in order to be able to differentiate the choice of specific forms of care: residential (formal) and informal. The SHARE data for Poland did not contain information on people using residential care, therefore, in order to achieve the purpose of the study, it was necessary to provide comparable information obtained directly from long-term care facilities. As no similar study was performed in Poland and there is no data available at the individual level on long-term care residents, we decided to collect unique data. At the stage of designing the research, we took care of the comparability of variables between the two databases. First, we used data collected by us in the years 2021–2022 on residents of inpatient LTC facilities (private rest homes, residential social homes and nursing homes). We sent out a questionnaire to the managers of institutions selected randomly from official registers kept by voivodeship offices and the Ministry of Health. Each type of facility is represented in all of the 16 voivodeships in Poland, and they vary in terms of the size of the place of their location. In the self-completion questionnaire, we asked for the data concerning selected socio-demographic information regarding health and independence, as well as family networks of all the residents. As a result, a unique database was created including 745 observations from the private rest homes, 2,258 observations from the residential social homes and 872 observations from the nursing homes. Another group of data was related to the people receiving informal care at home and those who do not receive any kind of care (no LTC). The data came from the SHARE, which is a biennial panel study conducted by using probability-based sampling on people aged 50 or older and their partners across European countries, including Poland [84, 85]. The data contains socio-demographic information about respondents as well as information on physical and mental health and functional capacity and received informal care. The presented analysis used data from wave eight, which was conducted in 2019/2020 [86, 87] and was limited to Poland (307 observations regarding informal care at home and 1,754 observations regarding no LTC). We used the information provided in the main questionnaire. As a result, the sample size of combined data from both databases was 5,936 observations in total.

Due to the necessity to make comparisons to SHARE, we decided to limit the sample to the age of 50+. From the SHARE database, regarding informal home care, we selected those who receive personal or domestic help (or both) at home, provided by members of the household or people outside the household. Regarding no LTC, we selected people who do not receive any kind of care^4^ (informal or formal). In our cross-sectional analysis we based on Andersen’s Behavioral Model [7] which allow us distinguish three group of factors classified into predisposing, enabling, and need factors. Logistic regression and multinomial logistic regression was used as an appropriate statistical model for categorical dependent variables.

We divided our econometric analysis into 3 stages. In the first stage we used logistic regression to compare the factors differentiating the people receiving some kind of LTC (informal care at home; or in private rest homes; or in social residential homes; or in nursing homes) from those who do not receive any kind of care. In the second stage, we used the multinomial logit to compare the recipients of informal care with those using in-patient care. This time, a dependent variable on four levels was used: inpatient care in private rest homes, social residential homes and in nursing homes. Informal care at home was used as the reference category for the comparison. In the third stage, the previously used multinomial logit was applied again, but this time separately among females and males.

Due to collinearity problem between the number of ADL limitations and particular ADL limitations, and also between the number of chronic diseases and particular chronic diseases, two versions of the model have been developed. In model 1, the number of ADL limitations and the number of chronic diseases were used, while in model 2 the type of ADL limitations and the type of chronic diseases were used.

In our models, we use the following three groups of factors considered at an individual level (see Table 1): *predisposing factors* (age, sex, education level), *enabling factors* (having a living partner, having living children, frequency of family’s members visits as a proxy for close relationships with family members or the involvement of family members in care, type of residence), *need factors* (functional health status – number of ADL limitations, type of ADL limitations, number of chronic diseases, type of diseases).

We are aware that among the variables it would be worth taking into account the income of the residents, or preferably the income of the family members (not only resident’s household) involved in the organization of care. As this data was not available for residents of long-term care facilities, the level of education in our study remains a proxy for the economic situation.

**Table 1:** List of variables used in explanatory analysis

Variables	Description
**Dependent**	
***Logistic regression***:	***2 levels***:
(1) no LTC	0 if no LTC;
(2) any kind of LTC	1 if any kind of LTC;
***Multinomial logistic regression***:	***4 levels***:
(3) informal care	1; reference category
Inpatient care:	
(4) private rest homes	2;
(5) social residential homes	3;
(6) nursing homes	4;
**Independent**	
*** Predisposing factors***:	
age	in ten years brackets: ≥ 50–60 (reference category); ≥60–70; ≥70–80; ≥80–90; ≥90
sex	1 - female, 0 - male;
education level	1 - primary education (reference category), 2 - secondary education, 3 - tertiary education;
*** Enabling factors***:	
having a living partner	1 if resident has a living partner or spouse, 0 - if doesn’t have;
having a living children	1 if resident has a living children or spouse, 0 - if doesn’t have;
frequency of family members visits	1 if resident has visited / helped at least once a week (frequency visits in case of in-patient care and frequency of receiving personal or domestic help in case of informal care), 0 - if less visited / helped;
size of resident’s place of living	1 - village (reference category), 2 - small town (less than 20k inhabitants), 3 - medium town (20k-100k inhabitants), 4 - big city (more than 100k inhabitants);
*** Need factors***	
number of ADL limitations	(0–5)
* Particular ADL limitations*:	
bathing	1- if resident has a bathing limitation; 0 - if doesn’t have;
dressing	1- if resident has a dressing limitation; 0 - if doesn’t have;
transferring	1- if resident has a transferring limitation; 0 - if doesn’t have;
feeding	1- if resident has a feeding limitation; 0 - if doesn’t have;
toileting or continence^a^	1- if resident has a toileting or continence limitation; 0 - if doesn’t have;
number of chronic diseases	(0–13)
* Particular chronic diseases*:	
Parkinson’s disease	1- if resident has a Parkinson’s disease; 0 - if doesn’t have;
heart diseases (incl. myocardial infarction)	1- if resident has a heart disease; 0 - if doesn’t have;
respiratory system diseases	1- if resident has a respiratory system disease; 0 - if doesn’t have;
hypertension	1- if resident has a hypertension; 0 - if doesn’t have;
diabetes	1- if resident has a diabetes; 0 - if doesn’t have;
chronic renal failure	1- if resident has a chronic renal failure; 0 - if doesn’t have;
depression	1- if resident has a depression; 0 - if doesn’t have;
Alzheimer or dementia diseases	1- if resident has a Alzheimer or dementia disease; 0 - if doesn’t have;
mental health problems - other	1- if resident has a other mental health problem; 0 - if doesn’t have;
cancer	1- if resident has a cancer; 0 - if doesn’t have;
vision impairment	1- if resident has a vision impairment; 0 - if doesn’t have;
hearing impairment	1- if resident has a hearing impairment; 0 - if doesn’t have;
other diseases (incl. stroke, somatic problems)	1- if resident has other diseases; 0 - if doesn’t have;

^a^Due to missing continence in the list of ADL limitations in SHARE data in comparing with 2021/2022 database of residents of LTC facilities we merged continence and toileting (it takes the value 1 – if toileting or continence limitation exists)

Source: Author’s elaborations

Differences between the two databases we used were noticeable in the case of 3 variables: frequency of visits by family members, ADL limitations, and chronic diseases. For the frequency of visits variable, we wanted to assess the degree of family involvement in care, so in the case of LTC facilities residents the proxy for this variable was the frequency of visits by family members, and in the case of informal care (SHARE database) the frequency of domestic and personal care received by family members.

In our questionnaire, we asked about the 6 ADLs using the Katz Index [4] (bathing, dressing, transferring, feeding, toileting, continence), while the SHARE questionnaire additionally listed getting into and out of bed but omitted continence. Hence, we decided to omit getting into and out of bed from the analysis and to combine toileting and continence, which took the value of 1 if any of these limitations occurred. The final number of ADL is therefore 5.

In addition, for chronic diseases, we did not use any available tool, which was dictated by the need to simplify our questionnaire as much as possible so that it could be easily completed by LTC staff. As a result of combining the databases, we did not use the original longer list of diseases, but only those that were the same or similar or that could be combined into specific, larger categories. As a result, we combined Alzheimer’s and dementia, as they occurred separately in our questionnaire and together in SHARE. In particular, it is worth mentioning how we combine the precise names of the diseases found in SHARE to the general categories we used in our questionnaire: lung disease such as chronic bronchitis or emphysema were categorized as respiratory system diseases; heart attack or myocardial infarction, coronary artery thrombosis or any other heart disease including congestive heart failure as heart diseases; other emotional disorders including fear, anxiety, nervous or psychiatric problems as other mental health problems; cataracts as vision impairment; having a hearing aid as hearing impairment. The cases of cancer might be underestimated regarding LTC facilities as we excluded hospice and palliative care facilities (in Poland hospice and palliative care is often reported separately from long-term care, however there are nursing homes dedicated to people suffering from cancer).

We did not follow any specific reporting tool as the questionnaire we designed had to be simplified as much as possible to encourage LTC staff to respond. However, regarding SHARE dataset, information about questionnaires, variable definitions and codes can be found in the SHARE Wave 8 methodology book [87]. All analyses were conducted using STATA 12.0.

## Results

### Descriptive statistics

Regarding the predisposing factors, the statistics^5^ of variables used in the explanatory analysis (see Table 2) show that females dominate in our sample for any type of LTC we studied, with the largest share of 74.42% observed in private rest homes, and the smallest in social residential homes – 54%). The informal care recipients are the youngest group of the older adults (mean of age is 74.65 years), while the oldest groups are observed in inpatient facilities (mean of age is respectively: 76.43 years in social residential homes, 79.92 years in nursing homes and 83.03 years in private rest homes). Among people staying in residential LTC facilities, those in private rest homes declare the highest level of education (53.33% – secondary education; 23% – tertiary education). In case of other types of care, the level of education is lower (the lowest number of people with secondary education is found in nursing homes – 34.32%, and with tertiary education in social residential homes – 5.12%). In terms of enabling factors, the highest proportion of people with a living partner (52.12%) and a child (92.25%) is observed among those who receive informal care at home, while for the residents of social residential homes these figures are the lowest (6.09% and 48.98% respectively). The residents of private rest homes and social residential homes are dominated by inhabitants of large cities (44.67% and 37.18% respectively), while people coming from rural areas prevail among the older adults in nursing homes and those who receive informal care (44.02% and 55.77% respectively). In terms of need factors, the highest level of dependency is observed among the residents of nursing homes (mean of number of ADL limitations is 3.66), while among informal care recipients it is at its lowest (mean of number of ADL limitations is 1.35). Regarding number of chronic diseases, the distribution is not obvious. Residents of social residential homes and informal care recipients suffer, on average, from 3.12 to 2.99 chronic diseases, while patients in nursing homes and private rest homes, respectively, from 2.28 to 1.71. Meanwhile, people who do not use any care have, on average, 1.72 chronic diseases (similar value as for private rest homes). This shows that the number of chronic diseases does not translate into the intensity of care, and that the type of disease is more important.

**Table 2:** Summary statistics of variables used in explanatory analysis (unweighted data)

	Inpatient private rest homes		Inpatient residential homes		Inpatient nursing homes		Informal home		No LTC	
	Obs.	%^b^/mean^c^	Obs.	%^b^/mean^c^	Obs.	%^b^/mean^c^	Obs.	%^b^/mean^c^	Obs.	%^b^/mean^c^
**Binary (=1) and category variables**										
**I. PREDISPOSING**										
Sex (Female)	739	74.42^b^	2165	54.00^b^	841	70.27^b^	307	60.59^b^	1754	55.07^b^
Primary education	110	23.66^b^	995	48.04^b^	259	59.27^b^	136	44.88^b^	457	26.07^b^
Secondary education	248	53.33^b^	970	46.84^b^	150	34.32^b^	144	47.52^b^	1106	63.09^b^
Tertiary education	107	23.01^b^	106	5.12^b^	28	6.41^b^	23	7.59^b^	190	10.84^b^
**II. ENABLING**										
Living partner	693	12.55^b^	2084	6.09^b^	681	16.45^b^	307	52.12^b^	1754	75.20^b^
Living children	721	85.99^b^	2068	48.98^b^	748	79.55^b^	271	92.25^b^	1437	92.35^b^
Frequency of visits ≥ once a week	686	26.97^b^	2004	7.93^b^	675	25.33^b^	243	65.43^b^	-	-
Place of living - village	146	19.95^b^	651	30.18^b^	320	44.02^b^	116	55.77^b^	530	48.49^b^
Place of living - small town	170	23.22^b^	381	17.66^b^	194	26.69^b^	21	10.10^b^	95	8.69^b^
Place of living - medium town	89	12.16^b^	323	14.97^b^	83	11.42^b^	37	17.79^b^	250	22.87^b^
Place of living - big city	327	44.67^b^	802	37.18^b^	130	17.88^b^	34	16.35^b^	218	19.95^b^
**III. NEED**										
ADL - Bathing	739	89.17^b^	2165	79.58^b^	841	86.33^b^	306	37.58^b^	1750	2.23^b^
ADL - Dressing	739	74.83^b^	2165	55.01^b^	841	81.09^b^	306	40.20^b^	1750	5.94^b^
ADL - Transferring	739	65.09^b^	2165	41.43^b^	841	77.76^b^	306	17.32^b^	1750	0.97^b^
ADL - Feeding	739	39.38^b^	2165	19.95^b^	841	45.30^b^	306	19.61^b^	1750	0.91^b^
ADL - Toileting or continence	739	74.56^b^	2165	48.45^b^	841	75.62^b^	306	20.59^b^	1750	1.14^b^
Parkinson disease	739	6.5^b^	2165	4.34^b^	841	4.64^b^	307	2.28^b^	1750	0.51^b^
Heart diseases (incl. myocardial infarction)	739	8.39^b^	2165	22.96^b^	841	13.20^b^	307	35.50^b^	1750	16.74^b^
Respiratory system diseases	739	3.65^b^	2165	6.93^b^	841	4.52^b^	307	12.70^b^	1750	5.77^b^
Hypertension	739	19.89^b^	2165	50.39^b^	841	47.32^b^	307	65.47^b^	1750	50.97^b^
Diabetes	739	12.31^b^	2165	19.49^b^	841	17.72^b^	307	25.08^b^	1750	19.60^b^
Chronic renal failure	739	3.79^b^	2165	7.16^b^	841	7.85^b^	307	6.51^b^	1750	1.89^b^
Depression	739	7.71^b^	2165	12.24^b^	841	12.01^b^	307	14.33^b^	1750	6.40^b^
Alzheimer or dementia diseases	739	48.85^b^	2165	42.22^b^	841	41.38^b^	307	10.75^b^	1750	1.43^b^
Mental health problems - other	739	8.66^b^	2165	35.57^b^	841	11.30^b^	307	14.66^b^	1750	6.74^b^
Cancer^a^	739	5.28^b^	2165	6.74^b^	841	0.36^b^	307	11.73^b^	1750	6.34^b^
Vision impairment	739	7.98^b^	2165	18.48^b^	841	16.65^b^	307	21.17^b^	1750	8.06^b^
Hearing impairment	739	14.34^b^	2165	16.03^b^	841	20.10^b^	307	9.45^b^	1750	4.23^b^
Other diseases (incl. stroke, somatic problems)	739	24.49^b^	2165	70.12^b^	841	31.63^b^	307	69.71^b^	1750	44.00^b^
**Continuous variables**										
**I. PREDISPOSING**										
Age	739	83.03^c^	2165	76.43^c^	839	79.92^c^	307	74.65^c^	1754	66.89^c^
**III. NEED**										
Numbers of ADL limitations	739	3.43^c^	2165	2.44^c^	841	3.66^c^	306	1.35^c^	1750	0.11^c^
Numbers of chronic diseases	739	1.71^c^	2165	3.12^c^	841	2.28^c^	307	2.99^c^	1750	1.72^c^

^a^Cancer might be not comparable between inpatient and informal care, because LTC facilities database was no included hospice and palliative care facilities

^b^Percentage of observations for which the value of the variable used in the explanatory analysis equals 1 (satisfies the condition)

^c^Mean value of variable used in explanatory analysis

Source: Authors’ own analysis based on the SHARE Wave 8 and data collected in 2021/2022 database of residents of LTC facilities

Given that, as mentioned earlier, the literature suggests that there is a close relationship between the level of education and health, and health inequalities caused by social factors are observed among older people in Poland [88], we decided to check our sample regarding the statistics of education level and place of living both for the presence of two or more chronic diseases (according to the full list) and limited to selected diseases^6^ and the number of ADL limitations (Additional file 1).

The results of our analysis show that differences in the percentage of the older adults who suffer from chronic diseases between care recipients and no LTC group are smaller than in case of ADL limitations between the same two groups. The statistics presented in Additional file 1 confirm the existence of health inequalities related to social status among people using LTC, but only regarding the number of chronic diseases. Among the older adults with higher education levels, the percentage suffering from two or more chronic diseases (in both variants) is, on average, lower than among those with primary and secondary education. The number of chronic diseases decreases as the level of education increases, both among the recipients of any form of care and among the people who do not use any care. As far as the place of residence is concerned, in the group of care recipients with primary and secondary education, the percentage of the older adults who suffer from two or more chronic diseases increases along with the increase in the size of the city. On the other hand, among people with higher education, this tendency is not observed. Regarding ADL limitations, in our sample we do not observe any correlation between level of education (share of care recipients with primary education is smaller than of those with tertiary education – 65.8% vs. 66.3%) and the size of place of living.

### Any kind of LTC vs. no LTC

Results of logistic regression regarding the first stage of our econometric analysis – the comparison between the older adults receiving any LTC with those who do not receive any kind of care – are presented in Table 3.

**Table 3:** Results of logistic regression (coefficients) – any kind of LTC vs. no LTC

	MODEL 1	MODEL 2
VARIABLES	Any kind of LTC vs. no LTC	Any kind of LTC vs. no LTC
**I. PREDISPOSING**		
Age 60–70	0.413**	0.529**
	(0.200)	(0.220)
Age 70–80	0.862***	1.004***
	(0.207)	(0.231)
Age 80–90	1.485***	1.563***
	(0.228)	(0.258)
Age ≥ 90	2.735***	2.663***
	(0.412)	(0.441)
Sex (Female)	-0.565***	-0.485***
	(0.123)	(0.137)
Secondary education	-0.0423	0.0373
	(0.140)	(0.157)
Tertiary education	-0.102	0.0296
	(0.236)	(0.258)
**II. ENABLING**		
Living partner	-1.719***	-1.376***
	(0.133)	(0.147)
Living children	-1.722***	-1.489***
	(0.162)	(0.179)
Small town	0.729***	0.585***
	(0.196)	(0.218)
Medium town	0.0512	-0.000912
	(0.172)	(0.189)
Big city	0.851***	0.858***
	(0.154)	(0.172)
**III. NEED**		
Number of ADL limitations	1.448***	
	(0.0845)	
Number of chronic diseases	0.270***	
	(0.0402)	
ADL - Bathing		3.288***
		(0.250)
ADL - Dressing		-0.444*
		(0.263)
ADL - Transferring		1.039**
		(0.452)
ADL - Feeding		0.814*
		(0.477)
ADL - Toileting or continence		1.286***
		(0.452)
Parkinson’s disease		1.157
		(0.714)
Heart diseases (incl. myocardial infarction)		0.0348
		(0.168)
Respiratory system diseases		0.0350
		(0.267)
Hypertension		-0.377***
		(0.140)
Diabetes		-0.351**
		(0.171)
Chronic renal failure		0.925**
		(0.413)
Depression		-0.727***
		(0.273)
Alzheimer or dementia diseases		2.060***
		(0.305)
Mental health problems - other		1.692***
		(0.221)
Cancer		0.439*
		(0.259)
Vision impairment		0.273
		(0.204)
Hearing impairment		0.288
		(0.256)
Other diseases (incl. stroke, somatic problems)		0.681***
		(0.137)
Constant	0.199	-0.355
	(0.231)	(0.260)
Observations	4,058	4,058
Pseudo R2	0.610	0.672
Prob > chi2	0	0

Standard errors in parentheses. Reference categories are: age group 50–60, primary education, village

Significance levels: * *p* < 0.1, ** *p* < 0.05, *** *p* < 0.01

Authors’ own analysis based on the SHARE Wave 8 and data collected in 2021/2022 database of residents of LTC facilities

In terms of the factors belonging to the predisposing group, we see that age is a factor that positively correlates with using any form of LTC (all age groups remain statistically significant, with the values ​​of the coefficients increasing as we move from younger to older age groups), which is consistent with other studies [27, 30, 31]. Being a woman negatively correlates with receiving LTC (which is surprising as LTC recipients are dominated by females due to their longer life on average). This result is in line with some studies [34], but opposite to other studies [27, 30]. The level of education is insignificant, although it would be expected that people with higher education experience better health for longer [50] and therefore are less likely to receive LTC.

As for the enabling factors, both having a living partner and a child negatively correlates with receiving LTC and this result is in line with a previous study [56, 57]. Most likely, this result can be explained by the fact that some people stay in LTC facilities due to loneliness [54, 55]. Place of residence also turned out to be statistically significant, although the results remain somewhat non-obvious. Compared to rural inhabitants, the older adults living in small towns and large cities are both much more likely to use any form of LTC, while living in a medium-sized city is insignificant. These results can be explained by the uneven distribution of LTC facilities in Poland, as well as the diversity of care patterns, depending on the size of the place of living. Seniors living in villages receive more help from family members than inhabitants of large cities [68]. Perhaps, therefore, the residents of smaller locations can more often count on support from informal care, and residents of larger cities from inpatient care.

In terms of need factors, it is observed that both the increase in number of ADL limitations and the number of chronic diseases goes hand in hand with using any care, consistent with appropriately previous studies [30, 31]. However, not all of the diseases we studied cause dependency. We can see that most of the chronic diseases remained insignificant (Parkinson’s disease, heart diseases, respiratory system diseases, vision impairment, hearing impairment), or even their impact was statistically significant but negative (hypertension, diabetes). On average, those who do not receive any care are more likely to suffer from hypertension and diabetes. The results show that diseases that make it impossible to function at home and are the main indication for care (apart from ADL limitations) are: chronic renal failure, Alzheimer or dementia diseases [31, 35], mental health problems, cancer, group of other diseases including stroke.

### Informal care vs. inpatient LTC

Table 4 presents the results of multinomial regression in case of informal care vs. inpatient LTC. In the group of predisposing factors, age turned out to be a strong predictor of using all three forms of inpatient care in relation to informal care. The influence remains statistically significant for the age group 70 + and grows for each subsequent age group. Thus, the results remained consistent with the previous research, where age was a strong predictor of institutionalization [30, 31]. Although the inpatient LTC sector is dominated by female residents, in relation to informal care, being a female negatively correlates with using inpatient forms of care [consistent with 34; but opposite to 30], which might be explained by the fact that informal care is dominated by females even more than institutional care. Having secondary and higher education, as opposed to primary education, positively correlates with the probability of using private rest homes only. This means that people with a better social (and presumably economic) status, whenever they have a choice, prefer to use private care rather than public care [53], as expected within the first hypothesis. However, it is worth noting here that educated people have higher incomes and they usually don’t qualify to stay in public facilities. Of course, it should be taken into consideration that the preferences of older people are mainly focused on home care, although it was not possible to include formal (paid) home care in this study.

**Table 4:** Results of multinomial logit (coefficients) – inpatient vs. informal care

	MODEL 1			MODEL 2		
VARIABLES	Private vs. Informal	Residential vs. Informal	Nursing vs. Informal	Private vs. Informal	Residential vs. Informal	Nursing vs. Informal
**I. PREDISPOSING**						
Age 60–70	0.617	0.676	0.400	0.715	0.833*	0.536
	(0.539)	(0.431)	(0.524)	(0.581)	(0.478)	(0.572)
Age 70–80	1.810***	1.315***	1.497***	2.121***	1.762***	1.781***
	(0.540)	(0.446)	(0.525)	(0.598)	(0.508)	(0.590)
Age 80–90	2.958***	1.587***	2.375***	3.067***	1.994***	2.402***
	(0.549)	(0.462)	(0.536)	(0.621)	(0.539)	(0.616)
Age ≥ 90	3.350***	1.818***	2.517***	3.097***	1.944***	2.155***
	(0.615)	(0.533)	(0.605)	(0.695)	(0.620)	(0.694)
Sex (Female)	-0.490*	-0.699***	-0.489*	-0.516*	-0.716***	-0.547*
	(0.263)	(0.235)	(0.268)	(0.299)	(0.271)	(0.306)
Secondary education	1.118***	0.259	0.227	1.333***	0.475*	0.308
	(0.267)	(0.237)	(0.266)	(0.305)	(0.276)	(0.305)
Tertiary education	1.883***	-0.0820	0.522	1.829***	-0.0348	0.553
	(0.467)	(0.450)	(0.493)	(0.526)	(0.503)	(0.548)
**II. ENABLING**						
Living partner	-1.905***	-2.205***	-1.291***	-1.629***	-2.002***	-1.057***
	(0.308)	(0.265)	(0.308)	(0.348)	(0.302)	(0.352)
Living children	-0.576	-1.855***	-0.918***	-0.378	-1.601***	-0.647*
	(0.355)	(0.326)	(0.356)	(0.381)	(0.352)	(0.382)
Frequency of visits ≥ once a week	-2.269***	-3.145***	-1.868***	-2.404***	-3.200***	-2.031***
	(0.272)	(0.246)	(0.275)	(0.314)	(0.286)	(0.316)
Small town	1.331***	1.142***	1.081***	1.026**	0.789**	0.939**
	(0.374)	(0.347)	(0.365)	(0.407)	(0.379)	(0.399)
Medium town	0.263	0.721**	-0.369	0.287	0.616*	-0.328
	(0.352)	(0.308)	(0.357)	(0.401)	(0.354)	(0.405)
Big city	1.598***	1.720***	-0.349	1.864***	1.835***	-0.0446
	(0.320)	(0.290)	(0.350)	(0.376)	(0.345)	(0.403)
**III. NEED**						
Number of ADL limitations	0.814***	0.569***	1.036***			
	(0.0750)	(0.0698)	(0.0785)			
Number of chronic diseases	-0.822***	-0.133**	-0.409***			
	(0.0764)	(0.0620)	(0.0713)			
ADL - Bathing				2.852***	2.676***	2.957***
				(0.426)	(0.383)	(0.449)
ADL - Dressing				-1.628***	-1.135***	-1.110**
				(0.482)	(0.433)	(0.495)
ADL - Transferring				0.741	0.664	2.165***
				(0.538)	(0.505)	(0.547)
ADL - Feeding				-1.052**	-1.501***	-0.892**
				(0.457)	(0.440)	(0.452)
ADL - Toileting or continence				2.608***	1.667***	1.642***
				(0.555)	(0.522)	(0.556)
Parkinson’s disease				0.915	0.871	0.185
				(0.872)	(0.843)	(0.885)
Heart diseases (incl. myocardial infarction)				-1.583***	-0.701**	-1.195***
				(0.329)	(0.281)	(0.322)
Respiratory system diseases				-0.617	-0.490	-0.766
				(0.492)	(0.417)	(0.498)
Hypertension				-1.809***	-0.629**	-0.463
				(0.295)	(0.267)	(0.294)
Diabetes				-0.667*	-0.366	-0.875**
				(0.348)	(0.312)	(0.351)
Chronic renal failure				0.440	0.819	1.509***
				(0.603)	(0.534)	(0.566)
Depression				-1.258***	-0.737**	-0.738*
				(0.429)	(0.371)	(0.417)
Alzheimer or dementia diseases				0.931**	1.255***	0.739*
				(0.383)	(0.367)	(0.386)
Mental health problems - other				-0.104	1.084***	0.138
				(0.365)	(0.322)	(0.361)
Cancer				-0.829*	-0.487	-3.093***
				(0.490)	(0.427)	(0.833)
Vision impairment				-1.024***	-0.544*	-0.146
				(0.368)	(0.320)	(0.351)
Hearing impairment				0.220	-0.0477	0.490
				(0.442)	(0.413)	(0.436)
Other diseases (incl. stroke, somatic problems)				-2.036***	-0.324	-2.114***
				(0.286)	(0.263)	(0.290)
Constant	-0.0576	3.053***	-0.179	-0.174	2.286***	-0.0592
	(0.597)	(0.492)	(0.586)	(0.650)	(0.550)	(0.654)
Observations	2,788	2,788	2,788	2,788	2,788	2,788
Pseudo R2	0.321	0.321	0.321	0.408	0.408	0.408
Prob > chi2	0	0	0	0	0	0

Standard errors in parentheses.Reference categories are: age group 50–60, primary education, village

Significance levels: * *p* < 0.1, ** *p* < 0.05, *** *p* < 0.01

Authors’ own analysis based on the SHARE Wave 8 and data collected in 2021/2022 database of residents of LTC facilities

As for the enabling factors, support networks are a significant factor, which correlates negatively with using all three forms of inpatient care as compared to informal care. Having a living partner shows the strongest negative impact in the case of social residential homes, which suggests that people staying there most often experience loneliness comparing to the residents of other inpatient care. In the presented model, having children also negatively correlates with using residential care, but when it comes to choosing private nursing homes, this effect was the weakest or statistically insignificant. These results suggest that having more children correlates with using social residential homes and nursing homes, but does not affect the choice in the case of private institutions. The more frequent (at least once a week) are the visits by family members (more frequent help with personal and domestic activities at home), the lower is the choice of using inpatient care rather than informal care [26]. The involvement of family members in care is therefore one of the most important factors limiting the use of formal residential care. On the one hand, this result could suggest that people with better-developed support networks (caring patterns focused on family care) less often become residents of inpatient LTC. This effect could also be caused by a positive relationship between networks and health. It is again noted that loneliness is conductive to institutionalization [54–57] .

In the case of the size of the place of residence, the results are inconclusive. Inhabitants of small towns up to 20,000 people are more likely to benefit from all forms of inpatient care compared to rural residents using informal care. Most likely, this is due to the differences in the possibilities of providing care between urban and rural residents, and perhaps the greater availability of residential care facilities in larger centers. In the case of medium-sized cities between 20 and 100 thousand, positive and statistically significant influence is observed only in the social welfare sector. The fact of living in a big city with over 100,000 inhabitants compared to people living in rural areas and receiving informal care, positively correlates with using both private and social residential homes, while it is statistically insignificant for nursing homes. Perhaps these results indicate the uneven distribution of residential LTC facilities depending on the size of the city, i.e. not all have equal access to the full offer of institutional care. Nevertheless, rural residents are less likely to become residents of inpatient LTC and use informal care most often [31, 67, 68].

In the case of the group of factors classified as need factors, undoubtedly the level of dependence (measured by the number of ADL limitations) is the strongest positive predictor of using inpatient care (for all three types) as compared to informal care [31, 35]. This means that people using informal care often remain more independent (and therefore do not require the involvement of informal caregivers so often). In model 2, where the impact of individual constraints was verified, the results also turn out to be inconclusive. Additional analyzes of placing particular ADL limitations individually in the model suggest that each limitation positively correlates with the use of inpatient care compared to informal care. However, when these variables are put together in the model, the correlation becomes negative in case of dressing and feeding, which may indicate that when the whole range of ADL limitations is considered, these specific activities do not require the use of inpatient care (informal caregivers are better at providing assistance in this type of activities and they are not an indication for placement in a inpatient facility). The positive impact of ADL-bathing or ADL-toileting or continence may be related to the fact that people staying in inpatient care facilities, regardless of the degree of independence in performing these activities, receive help on a routine basis.

Among the chronic diseases, only Alzheimer’s and dementia appear to positively correlate with using all three forms of inpatient care in a statistically significant way, as compared to informal care [35]. Other mental disorders positively correlate with going to social residential homes, while a chronic renal failure to nursing homes. The other diseases used in the study turned out to be either statistically insignificant or their influence on the use of inpatient LTC was negative. This means that mental disorders and diseases that seriously limit independent existence and have a direct impact on mental abilities, such as Alzheimer’s and dementia, are the most difficult types of diseases for informal caregivers (other diseases are not an indication for using institutional LTC).

The variable numbers of chronic diseases turn out to negatively correlate with the choice of each of the three analyzed types of inpatient care in relation to informal care. This means that Polish residents with a greater number of chronic diseases more often use care provided by family members or friends, as expected in the second hypothesis. In a way, this is a surprising result, as some literature [26] indicates a positive relationship between multimorbidity and the use of institutional LTC. But we can also find opposite results which show insignificance of multimorbidity on institutionalization [29]. The fact of a negative impact of multimorbidity on using inpatient care as compared to informal care may suggest the failure of the LTC system (for example problem with availability of inpatient LTC). On the other hand, as mentioned earlier, people with a lower socio-economic status (especially poorly educated) suffer from more number of chronic diseases and in a situation of limited access to public institutional care often opt for family care [52]. However, it is worth emphasizing that in our sample we have not found any relationship between the number of chronic diseases and the number of ADL limitations, which is consistent with another study [89] where showed that chronic diseases do not necessarily cause significant limitations in daily life.

### Informal care vs. inpatient LTC – differences between males and females

As gender may be a factor not only differentiating patterns of care but also the occurrence of chronic diseases and ADL limitations, additional models were conducted separately for females (Table 5) and males (Table 6). The results show differences between males and females in all three factor groups: predisposing, enabling and need. This gives support to the third hypothesis.

**Table 5:** Results of multinomial logit (coefficients) – inpatient vs. informal care for **females** only

	MODEL 1			MODEL 2		
VARIABLES	Private vs. Informal	Residential vs. Informal	Nursing vs. Informal	Private vs. Informal	Residential vs. Informal	Nursing vs. Informal
**I. PREDISPOSING**						
Age 60–70	1.502*	0.778	-0.0903	1.218	0.712	-0.228
	(0.836)	(0.580)	(0.726)	(0.913)	(0.679)	(0.823)
Age 70–80	3.023***	1.989***	1.700**	3.242***	2.544***	1.950**
	(0.843)	(0.608)	(0.720)	(0.941)	(0.730)	(0.845)
Age 80–90	4.382***	2.646***	2.864***	4.411***	3.168***	2.860***
	(0.846)	(0.623)	(0.722)	(0.966)	(0.772)	(0.872)
Age ≥ 90	4.100***	2.157***	2.320***	3.809***	2.361***	1.956**
	(0.889)	(0.674)	(0.774)	(1.016)	(0.829)	(0.931)
Secondary education	1.449***	0.224	0.190	1.915***	0.662	0.506
	(0.352)	(0.319)	(0.352)	(0.444)	(0.413)	(0.446)
Tertiary education	1.125*	-0.927	-0.355	1.100	-0.844	-0.273
	(0.595)	(0.572)	(0.624)	(0.713)	(0.683)	(0.735)
**II. ENABLING**						
Living partner	-2.294***	-2.046***	-1.776***	-2.282***	-2.065***	-1.766***
	(0.466)	(0.385)	(0.466)	(0.552)	(0.467)	(0.556)
Living children	-1.202**	-2.720***	-1.484***	-1.139*	-2.632***	-1.343**
	(0.556)	(0.526)	(0.557)	(0.622)	(0.592)	(0.623)
Frequency of visits ≥ once a week	-2.494***	-3.165***	-1.969***	-2.829***	-3.480***	-2.370***
	(0.364)	(0.337)	(0.366)	(0.447)	(0.418)	(0.449)
Small town	1.515***	1.453***	1.387***	1.321**	1.253**	1.428**
	(0.521)	(0.492)	(0.510)	(0.615)	(0.586)	(0.606)
Medium town	0.336	0.850**	-0.342	0.296	0.688	-0.306
	(0.451)	(0.404)	(0.456)	(0.548)	(0.495)	(0.550)
Big city	1.752***	2.070***	0.0678	1.806***	2.042***	0.0939
	(0.416)	(0.381)	(0.442)	(0.511)	(0.473)	(0.535)
**III. NEED**						
Number of ADL limitations	1.016***	0.769***	1.177***			
	(0.105)	(0.0994)	(0.109)			
Number of chronic diseases	-0.909***	-0.213***	-0.503***			
	(0.0965)	(0.0803)	(0.0912)			
ADL - Bathing				3.447***	3.076***	3.797***
				(0.588)	(0.531)	(0.622)
ADL - Dressing				-1.704**	-0.890	-0.995
				(0.664)	(0.604)	(0.667)
ADL - Transferring				0.319	0.334	1.886**
				(0.745)	(0.709)	(0.754)
ADL - Feeding				-1.229*	-1.724**	-1.081
				(0.687)	(0.671)	(0.681)
ADL - Toileting or continence				3.564***	2.434***	2.049***
				(0.782)	(0.740)	(0.775)
Parkinson’s disease				0.744	0.536	-0.322
				(1.258)	(1.222)	(1.285)
Heart diseases (incl. myocardial infarction)				-2.016***	-1.108***	-1.635***
				(0.455)	(0.402)	(0.444)
Respiratory system diseases				-1.438**	-0.545	-0.932
				(0.699)	(0.549)	(0.665)
Hypertension				-1.836***	-0.705*	-0.423
				(0.413)	(0.384)	(0.413)
Diabetes				-1.276***	-0.843**	-1.354***
				(0.453)	(0.409)	(0.452)
Chronic renal failure				0.873	1.128	1.505*
				(0.823)	(0.740)	(0.785)
Depression				-0.576	-0.118	-0.0638
				(0.555)	(0.493)	(0.548)
Alzheimer or dementia diseases				1.103**	1.261**	0.682
				(0.522)	(0.504)	(0.525)
Mental health problems - other				-0.456	0.585	-0.321
				(0.482)	(0.432)	(0.480)
Cancer				-0.805	-0.635	-2.802***
				(0.685)	(0.621)	(0.951)
Vision impairment				-1.056**	-0.435	-0.292
				(0.490)	(0.434)	(0.471)
Hearing impairment				-0.789	-0.903*	-0.198
				(0.583)	(0.545)	(0.574)
Other diseases (incl. stroke, somatic problems)				-2.517***	-0.666*	-2.561***
				(0.413)	(0.389)	(0.417)
Constant	-1.057	2.480***	0.0673	-0.522	2.260***	0.494
	(0.939)	(0.701)	(0.822)	(1.016)	(0.792)	(0.933)
Observations	1,701	1,701	1,701	1,701	1,701	1,701
Pseudo R2	0.335	0.335	0.335	0.434	0.434	0.434
Prob > chi2	0	0	0	0	0	0

Standard errors in parentheses. Reference categories are: age group 50–60, primary education, village

Significance levels: * *p* < 0.1, ** *p* < 0.05, *** *p* < 0.01

Authors’ own analysis based on the SHARE Wave 8 and data collected in 2021/2022 database of residents of LTC facilities

**Table 6:** Results of multinomial logit (coefficients) – inpatient vs. informal care for males only

	MODEL 1			MODEL 2		
VARIABLES	Private vs. Informal	Residential vs. Informal	Nursing vs. Informal	Private vs. Informal	Residential vs. Informal	Nursing vs. Informal
**I. PREDISPOSING**						
Age 60–70	-0.0631	0.495	0.617	0.641	1.095	1.167
	(0.801)	(0.687)	(0.811)	(0.902)	(0.795)	(0.910)
Age 70–80	0.597	0.545	0.958	1.346	1.304	1.554*
	(0.789)	(0.690)	(0.806)	(0.925)	(0.833)	(0.944)
Age 80–90	1.165	-0.253	1.082	1.792*	0.510	1.354
	(0.805)	(0.716)	(0.834)	(0.954)	(0.868)	(0.986)
Age ≥ 90	3.546***	1.923*	3.020**	3.443**	2.381*	2.655*
	(1.165)	(1.090)	(1.205)	(1.402)	(1.334)	(1.454)
Secondary education	0.373	0.358	0.443	0.668	0.770*	0.622
	(0.454)	(0.395)	(0.445)	(0.519)	(0.464)	(0.516)
Tertiary education	2.913***	1.201	2.066**	3.021***	1.329	2.270**
	(0.848)	(0.826)	(0.898)	(1.043)	(1.010)	(1.089)
**II. ENABLING**						
Living partner	-1.505***	-2.228***	-0.717	-1.348**	-2.108***	-0.575
	(0.451)	(0.399)	(0.457)	(0.525)	(0.472)	(0.540)
Living children	-0.255	-1.145**	-0.770	-0.0340	-0.881*	-0.521
	(0.516)	(0.462)	(0.519)	(0.585)	(0.530)	(0.589)
Frequency of visits ≥ once a week	-2.294***	-3.622***	-2.283***	-2.331***	-3.626***	-2.445***
	(0.464)	(0.408)	(0.474)	(0.557)	(0.503)	(0.576)
Small town	1.342**	0.871*	0.756	1.212*	0.539	0.676
	(0.583)	(0.528)	(0.563)	(0.639)	(0.585)	(0.625)
Medium town	-0.0879	0.432	-0.610	0.443	0.827	-0.148
	(0.622)	(0.515)	(0.618)	(0.737)	(0.634)	(0.736)
Big city	1.707***	1.367***	-0.993	2.463***	1.755***	-0.285
	(0.562)	(0.506)	(0.652)	(0.703)	(0.652)	(0.789)
**III. NEED**						
Number of ADL limitations	0.558***	0.318***	0.885***			
	(0.116)	(0.106)	(0.123)			
Number of chronic diseases	-0.751***	-0.00415	-0.216*			
	(0.139)	(0.106)	(0.122)			
ADL - Bathing				2.206***	2.448***	1.946**
				(0.726)	(0.660)	(0.777)
ADL - Dressing				-1.631**	-1.664**	-1.545*
				(0.814)	(0.722)	(0.873)
ADL - Transferring				1.140	0.783	2.278**
				(0.908)	(0.829)	(0.922)
ADL - Feeding				-1.411*	-1.711**	-1.140
				(0.773)	(0.725)	(0.757)
ADL - Toileting or continence				2.213**	1.543*	2.298**
				(0.906)	(0.843)	(0.928)
Parkinson’s disease				1.387	1.955	1.595
				(1.354)	(1.263)	(1.319)
Heart diseases (incl. myocardial infarction)				-1.606***	-0.736	-0.995*
				(0.587)	(0.495)	(0.583)
Respiratory system diseases				0.602	-0.114	-0.370
				(0.838)	(0.763)	(0.883)
Hypertension				-1.447***	-0.222	-0.276
				(0.511)	(0.454)	(0.512)
Diabetes				0.179	0.347	-0.135
				(0.750)	(0.700)	(0.762)
Chronic renal failure				-0.312	0.100	1.402
				(1.097)	(0.960)	(0.988)
Depression				-2.865***	-1.757**	-1.851**
				(0.901)	(0.721)	(0.840)
Alzheimer or dementia diseases				0.605	1.398**	1.144*
				(0.688)	(0.647)	(0.685)
Mental health problems - other				0.474	1.960***	0.871
				(0.732)	(0.658)	(0.715)
Cancer				-1.025	-0.0960	-13.89
				(0.931)	(0.777)	(330.4)
Vision impairment				-1.141*	-0.870	0.0898
				(0.676)	(0.588)	(0.640)
Hearing impairment				1.477*	0.913	1.310
				(0.873)	(0.840)	(0.875)
Other diseases (incl. stroke, somatic problems)				-1.391***	0.184	-1.453***
				(0.479)	(0.436)	(0.487)
Constant	0.924	3.262***	-0.610	-0.408	1.433*	-1.111
	(0.844)	(0.734)	(0.882)	(0.962)	(0.862)	(1.001)
Observations	1,087	1,087	1,087	1,087	1,087	1,087
Pseudo R2	0.324	0.324	0.324	0.422	0.422	0.422
Prob > chi2	0	0	0	0	0	0

Standard errors in parentheses. Reference categories are: age group 50–60, primary education, village

Significance levels: * *p* < 0.1, ** *p* < 0.05, *** *p* < 0.01

Authors’ own analysis based on the SHARE Wave 8 and data collected in 2021/2022 database of residents of LTC facilities

In the group of models where informal care at home is the reference category, in terms of predisposing factors, the first significant difference between the sexes can be seen in terms of age – the threshold among females is 70 + as opposed to 90 + among males. When it comes to education, there are also differences observed. The fact of having secondary education significantly positively correlates with using private rest homes only among females. Males with the same level of education are more likely to stay in social residential homes. As for females, higher education positively correlates with using private care only. In the case of males, higher education increases using both private forms of care and nursing homes.

For the enabling factors, having a living partner negatively correlates with utilization of all three forms of inpatient care only among females, while it is insignificant in case of nursing homes among males. Significant differences are also observed with regard to having a living child. This variable negatively correlates with receiving any type of residential care among females, while among males it is significant only in the case of social residential homes. The frequency of visits/help remains a variable that negatively correlates with receiving any kind of care. Hence, having a family, matters only if the family members are in close contact with the person who needs care. Living children are more likely to provide informal care for mothers than for fathers but it might be explained by the fact that females (especially spouses) most often play the role of informal caregivers [90, 91], hence males are more likely to receive informal care from their wives, but widowed females need to receive more support from their children. When it comes to the size of the place of residence, the greatest differences between the sexes occur in case of small towns, up to 20,000 inhabitants. Among females, living in small towns positively correlates with using inpatient care as compared to females in rural areas who are provided with informal care. Among males, this variable also positively correlates but only with private care and social residential homes and is insignificant for nursing homes. Inpatient care use patterns were similar for females and males living in large cities. This variable positively correlates with being residents of private rest homes and social residential homes but was insignificant for nursing homes.

In terms of the need factors, the direction of the impact of the variables, i.e.: number of ADL limitations (positive effect) and number of chronic diseases (negative effect) remains consistent among females and males. There are gender differences in the case of chronic diseases affecting the risk of using inpatient care compared to informal care. Heart diseases negatively correlate with using all three forms of inpatient care in the case of females compared to females receiving informal care (therefore, on average, females using informal care suffer from cardiovascular problems more often than females in inpatient care). When it comes to males, heart diseases negatively correlate with utilization of private rest homes and nursing homes. Among females, problems with the respiratory system also negatively correlates with using private care, while among males this variable remains insignificant. As for diabetes, on average, females receiving informal care at home suffer from this condition more often than females in all three types of inpatient care (the sign for this variable remains negative and statistically significant). Among males, this variable is insignificant. In the case of depression among females, this variable remains insignificant (depression is as common among females receiving informal care as among females in LTC facilities). Among males, depression negatively correlates with using inpatient care in all three analyzed types of inpatient care. This means that, on average, depression occurs more often among males staying at home than in LTC facilities (or this might be due to a different method of collecting data – data about no LTC and informal care groups came from direct interviews, and the data on residents were provided by the facilities’ staff). Alzheimer’s disease goes hand in hand with using residential care among both sexes, but the effect is statistically significant among females only for private care and social residential homes, and among males only for social residential homes and nursing homes. Regarding Alzheimer’s disease, there is therefore a gender differentiation according to the type of LTC facility. Other mental illnesses are statistically significant and positively correlate with using social residential homes only among males, while among females this variable is insignificant. There are also differences between males and females when it comes to hearing problems. Among males, it is a factor positively correlated with using private care, and among females, a factor that is insignificant or negatively correlated with using social residential homes. It therefore seems that informal care is more often provided to females despite of hearing problems, and in the case of males, hearing loss is a factor that increases the risk of institutionalization (still private rather than public).

## Discussion

In our study, based on the Andersen’s Behavioral Model of Health Services Use (1968) [7] we identified characteristics of people over the age of 50 that influence the probability of using different types of LTC in Poland compared to people who do not use any kind of LTC. We point out the factors that differentiate the choice between inpatient facilities as compared to informal care, and show the differences between sexes. All three hypotheses were confirmed.

We are aware that the level of income of all family members involved in providing care correlates with the choice of the form of care, however due to unavailability of this variable, we used an education level as a proxy for economic situation of older adults. The study confirms the first hypothesis that social inequalities influence decisions about the choice of LTC. Better educated people more often choose private care than people with a lower social status. Among the latter, the phenomenon of multi-morbidity (more than two chronic diseases) is more common, so social inequalities translate into inequalities in health. Therefore, it is important to both invest in education and develop the healthcare sector earlier in life. Such actions on the part of the government should mitigate the existing inequalities in health among the older adults.

However multi-morbidity is a predictor of using LTC to a limited extent. The influence of the number of chronic diseases depends on the variable used for comparisons. When we consider informal care vs. inpatient care the sign for multi-morbidity is negative, but when no LTC is used for the comparison with any kind of LTC, the sign is positive. This means that informal care beneficiaries suffer from more chronic diseases that residents of LTC facilities and no multi-morbidity itself, but particular diseases (especially Alzheimer’s, dementia and other mental diseases) should be taken into account when considering institutionalization, which confirms the second hypothesis. The number of ADL limitations is a much more relevant indicator, as it positively correlates with using LTC in each of the analyzed models.

We confirm existence of different patterns of LTC utilization between females and males with respect to all three groups of factors. Differences are observed regarding correlation between having a living partner and a child and institutionalization. Also we confirm the third hypothesis that there are differences between females and males in diseases that predisposed them to use LTC. Thus gender differences should be taken into account when planning future LTC arrangements.

Our results show that loneliness itself might be a strong predictor of social residential homes utilization. This observation is supported by two other results. Firstly, multi-morbidity is a factor with limited impact on shaping the demand for inpatient LTC. Secondly, for the older adults in social residential homes is noticed that number of ADL limitations is lower than for residents of other type of inpatient facilities. Thus, in the context of the public debate about the deinstitutionalization of the social LTC sector, our results suggest that in case of the older adults who stay in social residential homes because of their loneliness but without health reasons, there is a space to offer other type of LTC arrangements for example: housing estates for seniors. On the other hand, we also identified that Alzheimer’s disease, dementia or other mental health problems remain strong predictors of using social residential homes. For this group of the older adults it may be difficult or impossible to offer another form of care outside of institutional care. Therefore, it seems that the development of long-term psychiatric care and the promotion of behaviors that may delay the occurrence of Alzheimer’s and dementia from an early age are also the right direction to follow.

Our study has some limitations. Combining databases from two sources was a challenge for several reasons. The period of data collection, which coincided with the Covid-19 pandemic and the restrictions, may have influenced the underestimation of the ‘frequency of visits’ variable, even though in our survey we asked LTC staff to specify the visit frequency ‘usually’. In addition, the statistics presented should not be generalized to the whole population due to the impossibility of weighting the data dictated by different target populations and different way of drawing of the samples. The importance of ensuring maximum possible comparability regarding diseases and ADLs, meant that we were forced to drop some diseases or aggregate them into more general categories. Thus, the list of chronic diseases used in our analysis does not exhaust all possible types of diseases that the older adults suffer from. Therefore, there is a risk that we were not able to identify diseases other than those described, which would significantly increase the probability of institutionalization.

Also, it was not possible to include formal (paid) home care in this study, due to the lack of relevant data. Including this kind of care would allow us to extend the analysis, especially in the context of differences in care preferences depending on social status, as dependent people more often prefer to stay at their homes.

We are aware that, apart from demand factors, the decision-making selection should also take the supply factors (e.g. availability of facilities, price of stay, number of places, etc.) into account, but due to the comparability with SHARE data and objective difficulties in estimating the costs of informal care –at this stage we decided not to include the supply factors in the analysis. Probably the biggest deficit of the presented analysis is the lack of information on the economic situation of households of LTC residents, which was not available.

In addition, further analyzes should also use data from the households of dependent people, especially information on people directly involved in care (informal caregivers), as the decision-making processes related to the choice of the form of care are often collective decisions of households. The data collection methodology forced a specific selection of variables used in the model, hence the use of other methods of data collection – interviews with residents (often difficult due to the availability of people staying in inpatient facilities and / or poor health and difficulties in establishing contact) or asking questions about a hypothetical situation (the preferred form of care, if required) would certainly offer a broader perspective on the factors determining the selection of specific forms of care.

## Conclusions

In this study, based on the Andersen’s Behavioral Model of Health Services Use, we examined the relationship between predisposing, enabling and need factors on the use of long-term care in Poland. Combining data from SHARE Wave 8 and data collected in the 2021/2022 LTC resident database, we made a comparison between older adults (aged 50+) receiving any LTC with those who do not use any kind of care. We also made a comparison between users of informal care and users of three different types (nursing homes, social residential homes and private rest homes) of inpatient LTC. The results of our study indicated that social inequalities influence LTC choice decisions. However, multimorbidity is a predictor of LTC use to a limited extent. There are also differences among men and women correlating with the use of specific forms of LTC, indicating gender-dictated variation in patterns of care. Limitations of ADLs, Alzheimer’s disease, dementia and other mental illnesses as factors that increase the risk of institutionalization in particular should be considered in projections of future LTC sector development as well as providing implications for health policy.

### Supplementary Information

** Additional file 1: Table A1.** Statistics for education level and place of living vs. chronic diseases and ADL limitations

## Data Availability

The data under analysis has been obtained from the publicly available database SHARE: Survey of Health, Ageing and Retirement in Europe, http://www.share-project.org/data-access/user-registration.html. This paper uses data from SHARE Waves 8. The SHARE data collection has been funded by the European Commission, DG RTD through FP5 (QLK6-CT-2001-00360), FP6 (SHARE-I3: RII-CT-2006-062193, COMPARE: CIT5-CT-2005-028857, SHARELIFE: CIT4-CT-2006-028812), FP7 (SHARE-PREP: GA N°211,909, SHARE-LEAP: GA N°227,822, SHARE M4: GA N°261,982, DASISH: GA N°283,646) and Horizon 2020 (SHARE-DEV3: GA N°676,536, SHARE-COHESION: GA N°870,628, SERISS: GA N°654,221, SSHOC: GA N°823,782, SHARE-COVID19: GA N°101,015,924) and by DG Employment, Social Affairs & Inclusion through VS 2015/0195, VS 2016/0135, VS 2018/0285, VS 2019/0332, and VS 2020/0313. Additional funding from the German Ministry of Education and Research, the Max Planck Society for the Advancement of Science, the U.S. National Institute on Aging (U01_AG09740-13S2, P01_AG005842, P01_AG08291, P30_AG12815, R21_AG025169, Y1-AG-4553-01, IAG_BSR06-11, OGHA_04–064, HHSN271201300071C, RAG052527A) and from various national funding sources is gratefully acknowledged (see www.share-project.org). The datasets generated and analysed during the current study are not publicly available because there is no permission to share data from in-patient LTC facilities but data are available from the corresponding author on reasonable request with permission of in-patient LTC facilities. Statistical model syntax is available from one of the authors, Małgorzata Wrotek (mwrotek@wne.uw.edu.pl) on reasonable request.

## Footnotes

1 Authors’ calculations based on data from Eurostat - Population on 1 January by age group and sex [demo_pjangroup] access: July 30, 2022.

2 Authors’ calculations based on data from Eurostat – EUROPOP-19 - Demographic balances and indicators by type of projection [proj_19ndbi]; Baseline projections; access: July 30, 2022.

3 Including nursing homes, psychiatric nursing homes and psychiatric chronic medical care homes, hospices as well as palliative care wards.

4 This group included the people who received help with paper work (but did not receive any personal or domestic help), as we decided that it is not a good predictor of being independent and it concerned only 25 observations (1,4% of no LTC group).

5 Data was unweighted due to the combination of two databases, so statistics are specified for our sample and should not to be generalized onto whole population of LTC recipients.

6 Group of selected diseases includes: Parkinson disease, heart diseases (inc. myocardial infarction), respiratory system diseases, chronic renal failure, Alzheimer or dementia diseases, mental health problems – other, other diseases (incl. stroke, somatic problems).

## References

[1] Eurostat. Population on 1 January by age group and sex [DEMO_PJANGROUP]. Retrieved July 30., 2022, from: https://ec.europa.eu/eurostat/databrowser/product/view/DEMO_PJANGROUP?lang=en.

[2] Eurostat. Demographic balances and indicators by type of projection [PROJ_19NDBI]. Retrieved July 30., 2022, from: https://ec.europa.eu/eurostat/databrowser/product/view/PROJ_19NDBI?lang=en.

[3] OECD (2005). Long-term care for older people, the OECD Health Project.

[4] Katz S, Ford AB, Moskowitz RW, Jackson BA, Jaffe MW (1963). Studies of illness in the aged. The index of ADL: a standardized measure of biological and psychosocial function. J Am Med Association.

[5] Lawton MP, Brody EM (1969). Assessment of older people: self-maintaining and instrumental activities of daily living. Gerontologist.

[6] Stallard E. Long term care for aging populations. In International encyclopedia of public health. 2nd edition. Edited by: Quah S. San Diego: Academic Press; 2017. pp. 447–58. 10.1016/B978-0-12-803678-5.00256-3.

[7] Andersen RM. Families’ use of health services: a behavioral model of predisposing, enabling and need components. *PhD thesis*. Purdue University, West Lafayette, IN; 1968. https://docs.lib.purdue.edu/dissertations/AAI6902884/.

[8] Lamura G, Mnich E, Bien B, Krevers B, McKee K, Mestheneos L, Döhner H. Dimensions of future social service provision in the ageing societies of Europe. In VI European Congress of the International Association of Gerontology and Geriatrics. 2007. p. 5–8. https://scholar.google.com/scholar_lookup?title=Dimensions+of+future+social+service+provision+in+the+ageing+societies+of+Europe&conference=Proceedings+of+the+VI+European+Congress+of+the+International+Association+of+Gerontology.

[9] Nies H, Leichsenring K, Mak S. The Emerging Identity of Long-Term Care Systems in Europe. In *Long-Term Care in Europe*. Edited by: Leichsenring K, Billings J, Nies H. London. Palgrave Macmillan; 2013. 10.1057/9781137032348_2.

[10] European Commission. Health and long-term care in the European Union. Report. Special Eurobarometer. European Commission. ; 2007, 283/ Wave 67.3 – TNS Opinion & Social. https://sid-inico.usal.es/idocs/F8/FDO22761/health_european_union.pdf.

[11] Golinowska S. The system of long term care in Poland. *Enepri Research Report no. 83. Contribution to WP 1 of the ANCIEN Project*. Eur Netw Economic Policy Res Institutes; 2010 https://www.files.ethz.ch/isn/122415/Poland.pdf.

[12] Błędowski P, Maciejasz M (2013). Rozwój opieki długoterminowej w polsce – stan i rekomendacje [Development of long-term care in Poland - state and recommendations]. Nowiny Lekarskie.

[13] Jurek Ł (2007). Sektory opieki długoterminowej - analiza kosztów. [Long-term care sectors – cost analysis]. Gerontologia Polska [Gerontology Poland.

[14] Statistics Poland. Zdrowie i ochrona zdrowia w 2020 roku. [Health and health care in 2020]. ; 2021. Retrieved July 30, 2022, from https://stat.gov.pl/download/gfx/portalinformacyjny/pl/defaultaktualnosci/5513/1/11/1/zdrowie_i_ochrona_zdrowia_w_2020_aneks_tabelaryczny.xlsx.

[15] Ministry of Family and Social Policy. Report MRPiPS-05. ; 2020. Retrieved July 30, 2022 from https://www.gov.pl/attachment/3347bced-2ec1-4063-b5ca-181e3fe79aad.

[16] Local Data Bank (Statistics Poland), Retrieved July 30., 2022 from https://bdl.stat.gov.pl/bdl/dane/podgrup/tablica.

[17] Ustawa o pomocy społecznej z dnia. 12 marca 2004 r. – Dz.U. z 2018r., poz. 1508 [The Polish Social Assistance Act of March 12 2004]. https://isap.sejm.gov.pl/isap.nsf/download.xsp/WDU20040640593/U/D20040593Lj.pdf.

[18] Ustawa o świadczeniach opieki zdrowotnej finansowanych ze środków publicznych z dnia 27. sierpnia 2004 r., Dz. U. z 2021 r. poz. 1285 [The Polish Health Care Services Financed from Public Funds Act of August 27 2004]. https://isap.sejm.gov.pl/isap.nsf/download.xsp/WDU20042102135/U/D20042135Lj.pdf.

[19] Colombo F, Llena-Nozal A, Mercier J, Tjadens F (2011). Help wanted? Providing and paying for long-term care. OECD Health Policy Studies.

[20] Babitsch B, Gohl D, von Lengerke T. Re-revisiting Andersen’s Behavioral Model of Health Services Use: a systematic review of studies from 1998–2011. Psycho-social Med. 2012;9. 10.3205/psm000089. 10.3205/psm000089 PMC3488807 23133505

[21] Andersen RM (1995). Revisiting the behavioral model and access to medical care: does it matter?. J Health Soc Behav.

[22] Andersen RM, Davidson PL. Improving access to care in America: Individual and contextual indicators. In Changing the U.S. health care system: Key issues in health care policy and management. 3d edition. Edited by: Andersen RM, Rice TH, Kominski G. San Francisco: Jossey-Bass; 2007. p. 3–31. https://media.johnwiley.com.au/product_data/excerpt/44/07879852/0787985244.pdf.

[23] Shih CM, Wang YH, Liu LF, Wu JH (2020). Profile of Long-Term Care recipients receiving home and community-based services and the factors that influence utilization in Taiwan. Int J Environ Res Public Health.

[24] Travers JL, Hirschman KB, Naylor MD (2020). Adapting Andersen’s expanded behavioral model of health services use to include older adults receiving long-term services and supports. BMC Geriatr.

[25] Rahman M, Efird JT, Kendig H, Byles JE (2019). Patterns of home and community care use among older participants in the australian longitudinal study of women’s Health. Eur J Ageing: Social Behav Health Perspect.

[26] Zeng L, Xu X, Zhang C, Chen L. Factors Influencing Long-Term Care Service Needs among the Elderly Based on the Latest Anderson Model: A Case Study from the Middle and Upper Reaches of the Yangtze River. *Healthcare* 2019, 7(4): 157. 10.3390/healthcare7040157. 10.3390/healthcare7040157 PMC6955999 31816957

[27] Steinbeisser K, Grill E, Holle R, Peters A, Seidl H. (2018). Determinants for utilization and transitions of long-term care in adults 65 + in Germany: results from the longitudinal KORA-Age study. *BMC Geriatrics* 2018, 18(1): 172. 10.1186/s12877-018-0860-x. 10.1186/s12877-018-0860-x PMC6069853 30064373

[28] Chuang M-J. Home and Community-Based Long-Term Care Services in Taiwan: Factors and Effects Associated with the Utilization by Community Dwelling Dependent Elderly. *PhD thesis*. The Johns Hopkins University ProQuest Dissertations Publishing; 2017. http://jhir.library.jhu.edu/handle/1774.2/60227.

[29] Fu YY, Guo Y, Bai X, Chui EW (2017). Factors associated with older people’s long-term care needs: a case study adopting the expanded version of the Anderson Model in China. BMC Geriatr.

[30] Slobbe L, Wong A, Verheij RA, van Oers H, Polder JJ (2017). Determinants of first-time utilization of long-term care services in the Netherlands: an observational record linkage study. BMC Health Serv Res.

[31] Wu CY, Hu HY, Huang N, Fang YT, Chou YJ, Li CP (2014). Determinants of long-term care services among the elderly: a population-based study in Taiwan. PLoS ONE.

[32] Chen YM, Berkowitz B (2012). Older adults’ home-and community based care service use and residential transitions: a longitudinal study. BMC Geriatr.

[33] de Meijer CA, Koopmanschap MA, Koolman XH, van Doorslaer EK (2009). The role of disability in explaining long-term care utilization. Med Care.

[34] Thygesen E, Saevareid HI, Lindstrom TC, Nygaard HA, Engedal K (2009). Predicting needs for nursing home admission - does sense of coherence delay nursing home admission in care dependent older people? A longitudinal study. Int J Older People Nurs.

[35] Borrayo EA, Salmon JR, Polivka L, Dunlop BD (2002). Utilization across the continuum of long-term care services. Gerontologist.

[36] Wong A, Elderkamp-de Groot R, Polder J, van Exel J (2010). Predictors of long-term care utilization by dutch hospital patients aged 65+. BMC Health Serv Res.

[37] de Meijer CA. Studies of Health and Long-Term Care Expenditure Growth in Aging Populations. *PhD thesis*. Netspar Theses. Erasmus University Rotterdam; 2012. https://www.netspar.nl//assets/uploads/001_PhD_Claudine_de_Meijer.pdf.

[38] Bakx P. Determinants of Long-Term Care Use. *Master thesis*. Netspar Theses Erasmus University Rotterdam; 2010. https://www.netspar.nl/assets/uploads/MA_Pieter_Bakx_2010.pdf.

[39] Weaver FM, Weaver BA (2014). Does availability of informal care within the household impact hospitalisation?. Health Econ Policy Law.

[40] Wren MA, Normand C, O’Reilly D, Cruise S, Connolly S, Murphy C. *Towards the development of a predictive model of long-term care demand for Northern Ireland and the Republic of Ireland*. Centre for Ageing Research and Development in Ireland. Trinity College Dublin/Queen’s University Belfast; 2012. https://doras.dcu.ie/17967/.

[41] Mor V, Wilcox V, Rakowski W, Hiris J (1994). Functional transistions among the Elderly: patterns, predictors, and Related Hospital Use. Am J Public Health.

[42] Rockwood K, Stolee P, McDowell I (1996). Factors associated with institutionalization of older people in Canada: testing a multifactorial definition of frailty. J Am Geriatr Soc.

[43] Chen YM, Thompson EA (2010). Understanding factors that influence success of home and community-based services in keeping older adults in community settings. J Aging Health.

[44] Gruneir A, Bronskill SE, Poss JW, Older Women. A look at gender differences in health system use. In *Health System Use by Frail Ontario Seniors: An In-Depth Examination of Four Vulnerable Cohorts*. Edited by: Bronskill SE, Camacho X, Gruneir A, Ho MM. Toronto, ON: Institute for Clinical Evaluative Sciences; 2011. https://www.ices.on.ca/~/media/Files/Atlases-Reports/2011/Health-system-use-by-frail-Ontario-seniors/Full-report.ashx.

[45] Hirdes JP, Mitchell L, Maxwell CJ, White N (2011). Beyond the ‘iron lungs of gerontology’: using evidence to shape the future of nursing Homes in Canada. Can J Aging.

[46] Karp A, Kåreholt I, Qiu C, Bellander T, Winblad B, Fratiglioni L (2004). Relation of education and occupation-based socioeconomic status to incident Alzheimer’s disease. Am J Epidemiol.

[47] Kitagawa EM, Hauser PM (1973). Differential Mortality in the United States. A study in Socio-Economic Epidemiology.

[48] Valkonen T. Adult Mortality and Level of Education. A Comparison of Six Countries. In Health Inequalities in European Countries. Edited by: Fox J. Gower Publishing, Aldershot; 1989. pp. 142–62. https://www.scholar.google.com/scholar_lookup?title=Adult+mortality+and+level+of+education%3A+a+comparison+of+six+countries&publication_year=1987.

[49] Mackenbach JP, Kunst AE, Cavelaars AE, Groenhof F, Geurts JJ (1997). Socioeconomic inequalities in morbidity and mortality in western Europe. The EU Working Group on socioeconomic inequalities in Health. The Lancet.

[50] Buckley NJ, Denton FT, Robb AL, Spencer BG (2004). The transition from good to poor health: an econometric study of the older population. J Health Econ.

[51] Batljan I. Demographics and Future Needs for Public Long Term Care and Services among the Elderly in Sweden - The Need for Planning. *PhD thesis*. Stockholm Studies in Social Work, 24, Department of Social Work Stockholm University; 2007. https://www.diva-portal.org/smash/get/diva2:197046/FULLTEXT01.pdf.

[52] Portrait F, Lindeboom M, Deeg D (2000). The use of long-term care services by the dutch elderly. Health Econ.

[53] Bravo G, Dubois M, Dubuc N, Demers L, Blanchette D, Painter K, Lestage C, Corbin C (2015). Comparing the resident populations of private and public long-term care facilities over a 15-year period: a study from Quebec, Canada. Aging Soc.

[54] Garber AM, MaCurdy T. Predicting Nursing Home Utilization among the High Risk Elderly. *NBER Working Paper Series*. Cambridge, MA: National Bureau of Economic Research; 1989, 2843. 10.3386/w2843.

[55] Greene VL, Ondrich JI (1990). Risk factors for nursing home admissions and exits: a discrete-time hazard function approach. J Gerontol.

[56] Grundy E, Glaser K (1997). Trends in, and transitions to, institutional residence among older people in England and Wales:1971 to 1991. J Epidemiol Commun Health.

[57] Freedman VA (1996). Family structure and the risk of nursing home admission. J Gerontol B.

[58] Reynolds S. A Comparative Analysis of Long-term Care Policies and Placements. *Master thesis*. Institute of Health Policy, Management and Evaluation University of Toronto; 2013 https://ace.ihpme.utoronto.ca/wp-content/uploads/disertations/msc-2013-17.pdf.

[59] de Meijer CA, Koopmanschap M, Bagod’Uva T, van Doorslaer E. Time To Drop – Time-To-Death? – Unravelling The Determinants of LTC Spending In the Netherlands. *Health Econometrics and Data Group Working Paper*. University of York; 2009, 09/33. https://www.york.ac.uk/media/economics/documents/herc/wp/09_33.pdf.

[60] Headen AE (1993). Economic disability and health determinants of the hazard of nursing home entry. J Hum Resour.

[61] Nihtilä E, Martikainen P (2007). Household income and other socio-economic determinants of long-term institutional care among older adults in Finland. Popul Stud.

[62] Grundy E, Jitlal M (2007). Socio-demographic variations in moves to institutional care 1991–2001: a record linkage study from England Wales. Age Ageing.

[63] Hancock R, Arthur A, Jagger C, Matthews R (2002). The Effect of Older People’s Economic Resources on Care Home Entry under the United Kingdom’s Long-Term Care Financing System. J Gerontol B.

[64] Breeze E, Sloggett A, Fletcher A (1999). Socioeconomic and demographic predictors of mortality and institutional residence among middle aged and older people: results from the Longitudinal Study. J Epidemiol Commun Health.

[65] Grundy E (1992). Socio-demographic variations in rates of movement into institutions among elderly people in England and Wales: an analysis of linked census and mortality data 1971–1985. Popul Stud.

[66] Glaser K, Grundy E, Lynch K (2003). Transitions to supported environments in England and Wales among elderly widowed and divorced women: the changing balance between co-residence with family and institutional care. J Women Aging.

[67] McCann M, Grundy E, O’Reilly D (2014). Urban and rural differences in risk of admission to a care home: a census-based follow-up study. Health Place.

[68] Błędowski P. Potrzeby opiekuńcze. [Care needs]. In *Badanie poszczególnych obszarów stanu zdrowia osób starszych, w tym jakości życia związanej ze zdrowiem*. [*Research on specific areas of the aging adults’ health condition, including health-related quality of life*]. Edited by: Błędowski P, Grodzicki T, Mossakowska M, Zdrojewski T. Medical University of Gdańsk; 2021: 913–929.

[69] Kramer M (1980). The rising pandemic of mental disorders and associated chronic diseases and disabilities. Acta Psychiatrica Scandinavica..

[70] Gruenberg EM (1977). The failures of success. *The Milbank Memorial Fund Quarterly*. Health and Society.

[71] Olshansky SJ, Rudberg MA, Carnes BA, Cassel CK, Brody JA (1991). Trading off longer life for worsening health: the expansion of morbidity hypothesis. J Aging Health.

[72] Fries JF (1980). Aging, natural death, and the compression of morbidity. N Engl J Med.

[73] Manton KG (1982). Changing Concepts of Morbidity and Mortality in the Elderly Population. Milbank Meml Fund Q Health Soc.

[74] Fries JF (2003). Measuring and monitoring success in compressing morbidity. Ann Intern Med.

[75] Spillman BC (2004). Changes in elderly disability rates and the implications for health care utilization and cost. Milbank Q.

[76] Freedman VA, Crimmins EM, Schoeni RF, Spillman BC, Aykan H, Kramarow E, Land K, Lubitz J, Manton K, Martin LG, Shinberg D, Waidmann T (2004). Resolving inconsistencies in trends in old-age disability: report from a technical working group. Demography.

[77] Costa DL (2000). Understanding the Twentieth-Century decline in chronic conditions among older men. Demography.

[78] Christensen K, Doblhammer G, Rau R, Vaupel JW. Ageing populations: the challenges ahead. *The Lancet* 2009, 374(9696): 1196 – 208. 10.1016/S0140-6736(09)61460-4. 10.1016/S0140-6736(09)61460-4 PMC2810516 19801098

[79] Shugarman LR, Fries BE, James M (1999). A comparison of home care clients and nursing home residents: can community based care keep the elderly and disabled at home?. Home Health Care Serv Q.

[80] Gaugler JE, Duval S, Anderson KA, Kane RL. Predicting nursing home admission in the U.S: a meta-analysis. BMC Geriatr. 2007;7(13). 10.1186/1471-2318-7-13. 10.1186/1471-2318-7-13 PMC1914346 17578574

[81] Cohen-Mansfield J, Wirtz PW. The reasons for nursing home entry in an adult Day Care Population: Caregiver Reports Versus Regression results. J Geriatr Psychiatr Neurol *2009*, 22(4): 274–81. 10.1177/0891988709335799. 10.1177/0891988709335799 19429847

[82] Nihtilä EK, Martikainen PT, Koskinen SV, Reunanen AR, Noro AM, Häkkinen UT (2008). Chronic conditions and the risk of long-term institutionalization among older people. Eur J Pub Health.

[83] Connolly S, O’Reilly D (2009). Variation in care home admission across areas of Northern Ireland. Age Ageing.

[84] Börsch-Supan A, Brandt M, Hunkler C, Kneip T, Korbmacher J, Malter F, Schaan B, Stuck S, Zuber S (2013). Data Resource Profile: the Survey of Health, Ageing and Retirement in Europe (SHARE). Int J Epidemiol.

[85] Börsch-Supan A, Jürges H (2005). The Survey of Health, Ageing and Retirement in Europe – Methodology.

[86] Börsch-Supan A. *Survey of Health, Ageing and Retirement in Europe (SHARE) Wave 8*. Release version: 8.0.0. SHARE-ERIC. Data set; 2022. 10.6103/share.w8.800.

[87] Bergmann M, Börsch-Supan A (2021). SHARE Wave 8 methodology: collecting cross-national Survey Data in Times of COVID-19.

[88] Lange A, Zdrojewski T, Zagożdżon P, Błędowski P, Jagiełło K, Wizner B, et al. Nierówności w zdrowiu w zależności od czynników społecznych. [Health inequalities depending on social factors]. In Badanie poszczególnych obszarów stanu zdrowia osób starszych, w tym jakości życia związanej ze zdrowiem. [Research on specific areas of the aging adults’ health condition, including health-related quality of life]. Edited by: Błędowski P, Grodzicki T, Mossakowska M, Zdrojewski T. Gdańsk, Medical University of Gdańsk; 2021. pp. 1035–52. https://polsenior2.gumed.edu.pl/attachment/attachment/82370/Polsenior_2.pdf.

[89] Guralnik JM (1996). Assessing the impact of comorbidity in the older population. Ann Epidemiol.

[90] Johansson MF, McKee KJ, Dahlberg L, Williams CL, Summer Meranius M, Hanson E, Magnusson L, Ekman B, Marmstål Hammar L (2021). A comparison of spouse and non-spouse carers of people with dementia: a descriptive analysis of swedish national survey data. BMC Geriatr.

[91] Sharma N, Chakrabarti S, Grover S (2016). Gender differences in caregiving among family - caregivers of people with mental illnesses. World J Psychiatry.

