# Supplementary material for: Predictors of long-term care use - informal home care recipients versus private and public facilities residents in Poland
Source: BMC Geriatr. 2023 Aug 24;23:512. doi: 10.1186/s12877-023-04216-2 (PMC10463875; doi:10.1186/s12877-023-04216-2)
Supplement: Supplementary file 1 — Additional file 1: Table A1. Statistics for education level and place of living vs. chronic diseases and ADL limitations [file 12877_2023_4216_MOESM1_ESM.docx]

Table A1 Statistics for education level and place of living vs. chronic diseases and ADL limitations.

|  | 2 or more chronic diseases (all) | | | 2 or more chronic diseases (selected) | | | 2 or more ADL | | |
| --- | --- | --- | --- | --- | --- | --- | --- | --- | --- |
|  | inpatient and informal LTC | no LTC | total sample | inpatient and informal LTC | no LTC | total sample | inpatient and informal LTC | no LTC | total sample |
| **EDUCATION LEVEL** | | | | | | | | | |
| Primary education | 76.7 | 60.5 | 72.9 | 51.4 | 23.5 | 44.9 | 65.8 | 4.8 | 51.6 |
| Secondary education | 75.5 | 48.4 | 64.1 | 49.8 | 14.1 | 34.8 | 59.5 | 1.5 | 35.0 |
| Tertiary education | 69.7 | 36.8 | 56.0 | 42.8 | 13.2 | 30.4 | 66.3 | 0.5 | 38.8 |
| **PLACE OF LIVING** | | | | | | | | | |
| Village | 71.0 | 50.0 | 64.7 | 42.9 | 18.5 | 35.6 | 68.3 | 2.3 | 48.4 |
| Small town | 71.2 | 57.9 | 69.7 | 44.3 | 16.8 | 41.2 | 74.7 | 4.2 | 66.9 |
| Medium town | 75.6 | 51.6 | 67.9 | 48.3 | 18.0 | 38.6 | 65.8 | 2.8 | 45.7 |
| Big city | 74.7 | 46.8 | 70.7 | 52.0 | 12.8 | 46.3 | 63.7 | 2.8 | 54.9 |

Source: Authors’ own analysis based on the SHARE Wave 8 and data collected in 2021/2022 database of residents of LTC facilities
